# Comparative Proteomic Analysis of Tumor Mesenchymal-Like Stem Cells Derived from High Grade versus Low Grade Gliomas

**DOI:** 10.22074/cellj.2016.4179

**Published:** 2017-02-22

**Authors:** Mousa Taghipour, Aydine Omidvar, Mahboobeh Razmkhah, Abbas Ghaderi, Zahra Mojtahedi

**Affiliations:** 1 Department of Neurosurgery, Faculty of Medicine, Shiraz University of Medical Sciences, Shiraz, Iran; 2 Shiraz Institute for Cancer Research, School of Medicine, Faculty of Medicine, Shiraz University of Medical Sciences, Shiraz, Iran

**Keywords:** Glioma, Mesenchymal Stem Cells, Proteomics, Pyruvate Kinase

## Abstract

**Objective:**

Gliomas are the most common primary brain tumors, and have been ranked as the fourth leading cause of cancer death. Tumor mesenchymal-like stem cells (tMSCs) contribute to the aggressive behavior of glial tumors by providing a favorable microenvironment for the malignant cells. The aim of our study was to identify differential proteome of tMSCs derived from low vs. high grade glioma tumors.

**Materials and Methods:**

Patients with newly diagnosed low and high grade gliomas were included in this case control study. tMSCs were isolated from tumors using enzymatic digestion validated by flow cytometer analysis after sub-culturing. Differential proteomic analysis of tMSCs derived from low and high grade tumors was performed by two-dimensional gel electrophoresis and mass spectrometry. Protein spots with more than two fold differences and P values below 0.05 were considered as differentially expressed ones.

**Results:**

In tMSCs isolated from low and high grade gliomas, different isoforms of mesenchymal-related proteins vimentin and transgelin were differentially expressed. Overexpressed proteins in tMSCs isolated from low grade gliomas were mitochondrial manganese-containing superoxide dismutase (Mn-SOD), 40S ribosomal protein SA, and GTP-binding nuclear protein, while in tMSCs isolated from high grade gliomas, cathepsin B, endoplasmin, ezrin, peroxiredoxin1, and pyruvate kinase (PK) were found to be significantly overexpressed.

**Conclusion:**

For the first time, we analyzed the differential proteomic profiles of tMSCs isolated from glioma tumors with different grades. It was found that molecules related to mesenchymal cells (vimentin and transglin), in addition to those related to tumor aggressiveness with potential secretory behavior (e.g. cathepsin B) were among differentially expressed proteins.

## Introduction

Malignant brain tumors comprise a small percentage of all tumors. Their incidence is about 4-5 in 100,000 adult per year; however, their malignant nature has made them the fourth leading cause of cancer death (1). Glial cells, a group of non-neuronal cells, provide support for neurons, and may be transformed to distinct central nervous system (CNS) neoplasms depending on the transformed glial cell type. Glial tumors arising from astrocytes are the most common primary CNS neoplasm (2). The term of glioma is frequently referred to astrocytoma to exclude other types of glial tumors. Gliomas are graded from I to IV. Grades I and II are referred to low-grade gliomas. These tumors are circumscribed and well-differentiated. They are also characterized by a mild to moderate increase in the number of glial cell nuclei. Grade III is referred to anaplastic astrocytoma in which more densely cellular regions and higher nuclear pleomorphism are observed. Mitotic figures are also present. Glioblastoma multiforme (GBM) is referred to grade IV of the tumor. GBM has a histological appearance similar to that of anaplastic astrocytoma, in addition to the presence of either necrotic areas or extensive new blood vessel formation. In terms of prognosis, there is a substantial difference between grades I/II and III/IV (2,3). Approximate survival is eight years for a low- grade glioma (grade I or II). The survival is reduced to two to three years for an anaplastic astrocytoma, and around 1 year for a GBM despite aggressive treatment including resection combined with radiotherapy and chemotherapy. While GBMs constitute two-thirds of all gliomas, anaplastic astrocytomas and low-grade gliomas constitute two-thirds and one-third of the rest, respectively (2). 

Progression of cancers, including gliomas, is facilitated by its environment comprising a variety of cells, notably mesenchymal-like stem cells (MSCs) (4). Recently, endogenous tumor MSCs (tMSCs) 

were purified from GBM. These GBM-derived tMSCs do not form tumors upon transplantation showing that tMSCs are not a transdifferentiated tumor cell type, but indicate that tMSCs are part of the natural GBM microenvironment (4,5). Proteomic profiling represents the large-scale analysis of protein expression and post-translational modifications of proteins in tissues. Through comparative proteomic profiling of different biological materials, pathologic molecular alterations leading to tumorigenesis can be discovered. In addition, proteomics showed to be promising for detection of potential diagnostic, prognostic and treatment-assessing biomarkers in a variety of disease (6,7). Identification of molecular markers of tMSCs in glioma can shed light on the pathogenesis of this malignancy, and eventually lead to the control of the disease in a more efficiently way. 

In the present study, we cultured stem like cells from tumor tissues obtaining from patients with intracranial low and high grade gliomas. For the first time, we analyzed the comparative proteomic profile of these cells by two dimensional electrophoresis (2DE) 

as well as mass spectrometry, and identified the overexpressed proteins in these cells. 

## Materials and Methods

This case control study was approved by the Ethics Committee of Shiraz University of Medical Sciences (Shiraz, Iran), and informed consent was obtained from all patients. tMSCs were isolated from the tissue samples obtained from 9 patients with different grades of intracranial glioma. Demographic information of patients is shown in Table 1. 

**Table 1:** Demographic information, symptoms, signs, location of tumors and pathologic diagnosis in each patient


Case	Age (Y)	Sex	Location of symptoms	Signs	Pathologic diagnosis

1	61	F	Lt temporal	HARecent memory lossSensory dysphasia	GBM (grade IV)
2	27	F	Lt temporoparietal	HABlurred vision Partial seizurePapilledema	Astrocytoma (grade II)
3	67	M	Rt frontal	Lt side hemiparesisGTCSPapilledema	GBM (grade IV)
4	29	M	Rt frontal	HALt side hemiparesisGTCS	Oligoastrocytoma (grade II)
5	33	F	Lt frontal	HABlurred visionPapilledema	GBM (grade IV)
6	27	F	Lt frontal	HABlurred visionPapilledema	Astrocytoma (grade II)
7	36	M	Lt frontal	GTCSPapilledema	GBM (grade IV)
8	38	M	Rt frontotemporal	Complex partial seizureGTCS	Astrocytoma (grade II)
9	63	M	Lt frontal	HAGTCS	Anaplastic oligodendroglioma (grade III)


HA; Headache, F; Female, Lt; Left, M; Male, Rt; Right, and GBM; Gliobastoma multiforme, GTCS; Generalized tonic-clonic seizures.

**Fig.1:**
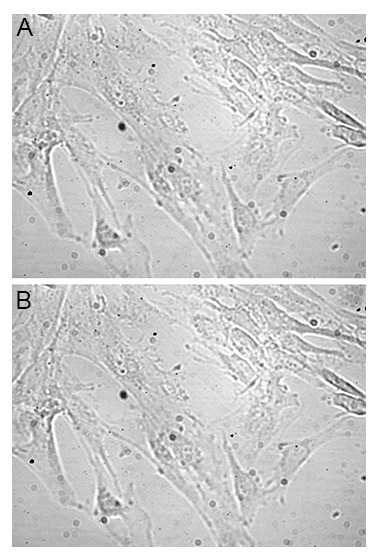
Microscopic views of mesenchymal-like stem cells derived from A. Low and B. High grade gliomas.

The glioma tissue was obtained from each patient in small pieces, digested enzymatically, and centrifuged. The pellet containing the adherent cells was resuspended in Dulbecco’s Modified Eagle Medium (DMEM, Biosera, USA) with 10% fetal bovine serum (Gibco, USA) and 1% penicillin/ streptomycin (Biosera, UK). Non-adherent cells were discarded 48 hours post-culture. The adherent cells were cultured until they reached confluence. They were then trypsinized and seeded at a density of 5×10^3^ cells/cm^2^ . They were finally harvested after 30 days of culture in order to produce a homogenous population of cells. Isolated cells were stained for the expression of mesenchymal markers CD44, CD105 and CD166 (BD Biosciences, USA), and lack of expression CD45 and CD34. Microscopic view of the isolated cells, which were the spindle shape, adherent and compatible with mesenchymal cells are shown in Figure 1. 

For 2DE experiment, the cultured cells were harvested by trypsinization and washed three times with cold phosphate-buffered saline (PBS, Sigma, Germany) and centrifuged. Pelleted cells were lysed with lysis buffer containing 7 M urea, 2 M Thiourea (Merck, Germany), 4% w/v 3-[(3-Cholamidopropyl) dimethylammonio]-2-hydroxy-1-propanesulfonate (CHAPS), GE Healthcare, 40 mM dithiothreitol (DTT), 2% vol/vol immobilized pH gradient (IPG) 3-10 buffer (Uppsala, Sweden). After centrifugation, the supernatant was stored at -70˚C. Protein concentration was determined by Bradford method. 2DE experiment was performed according to the previously described protocol (6). Briefly, first dimension isoelectric focusing (IEF) was performed using the PROTEAN IEF Cell (Bio-Rad, USA). About 500 μg of protein extracts from each sample was loaded per immobilized pH gradient (IPG) strip (pH=3-10 NL, GE Healthcare, UK). Then IEF was carried out at 20˚C with focusing at 10,000 V for a total of 70,000 Volt hour. 

After IEF, strips were equilibrated, and subsequently the strips were sealed with 0.5% agarose on top of a 12% acrylamide gradient gel (180×200×1.5 mm) with a constant current of 15 mA/gel for 10 minutes, followed by current of 30 mA/gel. The gels were visualized with colloidalCoomassieBrilliant Blue G-250, and scanned using GS-800 Imaging Densitometer (Bio-Rad, USA) at 300 dpi resolution. 

The images were classified into two groups (low vs. high grade gliomas) and analyzed by the Image Master 2D Platinum software, version 7 (Swiss Institute of Bioinformatics, Switzerland) according to the manufacturer’s instructions. Spots with differences in normalized spot volume (vol%) greater than two fold between low and high grade gliomas were subjected to further statistical analyses using t test and Mann-Whitney test. P values of below 0.05 were considered as statistically significant different. Spot groups with more than two fold differences and P values below 0.05, were initially considered as differentially expressed protein spots. The presence and overexpression of these differentially expressed spots were validated by eye in at least three images in each group before sending for mass spectrometry (MS) identification. 

Differentially expressed protein spots in at least three patients were cut from the gel using a pipette tip, and transferred into microcentrifuge tubes (Eppendorf, Germany). Then, the spots were sent to the Bioscience Technology Facility, Department of Biology, University of York (York, UK) for trypsin in gel digestion and MALDI-TOF/TOF-MS analysis. MALDI-TOF/TOF-MS was performed using a Brukerultraflex III MALDI-TOF/TOF (BrukerDaltonics, Germany). 

## Results

**Fig.2:**
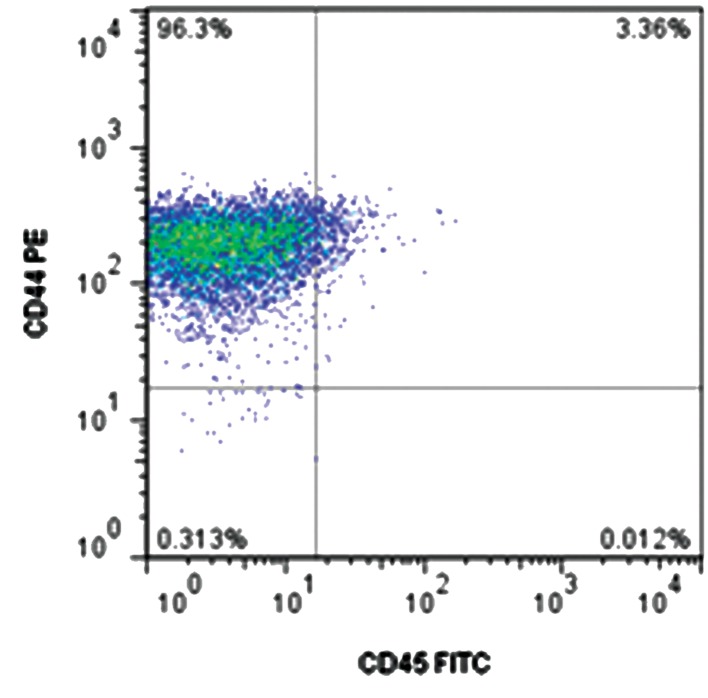
Flow cytometer representative of CD44 and CD45 expressions in tumor mesenchymal-like stem cells.

In the present study, we isolated MSCs from low and high grade gliomas. Both morphology (Fig .1A,B) and their markers proved their mesenchymal properties. Flow cytometer analysis revealed that mesenchymal-like cells were positive 96 ± 1.7, 85 ± 3.5, and 95 ± 2 in low grade glioma, and 95 ± 1, 69 ± 2.5, and 82 ± 7 in high grade glioma for CD44, CD105 and CD166, respectively. The expressions of CD34 and CD45 were lower than 1% in all isolated cells. A representative image of expressions for CD44 and CD45 was shown in Figure 2. The isolated cells were lysed and subjected to 2DE. Differentially expressed protein spots were picked up from gels and sent for identification by MS. 

**Fig.3:**
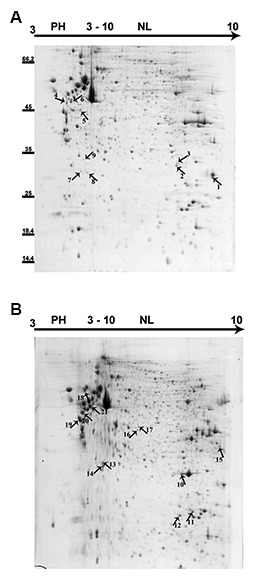
Representatives of two dimensional gels related to mesenchymal-like stem cells derived from A. Low and B. High grade gliomas. 21 differentially expressed protein spots were identified by mass spectrometry. The spot details are represented in Table 2.

Collectively 22 reproducible, distinct and intense spots were picked up from gels. Of 22 spots, 21 were identified by MS. As shown in Figure 3, in the gels obtained from low grade tMSCs, the identified proteins were five isoforms of vimentin (with different molecular weight and isoelectric point), transgelin, mitochondrial manganese-containing superoxide dismutase (Mn-SOD), GTP-binding nuclear protein and 40S ribosomal protein SA. In the gels obtained from high grade tMSCs, the identified proteins were three isoforms of vimentin, two isoforms of transgelin, mithochondrial superoxide dismutase and a single peptide match to peroxiredoxin1, two isoforms of cathepsin B, pyruvate kinase (PK), endoplasmin, a single peptide match to splicing factor/proline- glutamine rich and ezrin. The descriptions of the identified protein spots are summarized in Table 2. 

**Table 2:** Mass spectrometry identification of protein spots extracted from gels of low grade (spot numbers 1-9) versus high grade tumors (spot numbers: 10-21). Spot numbers are similar the same as in Figure 1


Spot no.	Protein name	Molecular wight (theor)	pI (theor)	Score^a^	Matched peptides	Accession no.		Sequence-coverage (%)	

1	Transgelin	22.6	8.87	180	4	Q01995		16	
2	Mitochondrial manganese-containing superoxide dismutase (Mn-SOD)	24.8	8.35	131	2	P04179		13	
3	GTP-binding nuclear protein Ran	24.5	7.01	73	2	P62826		8	
4	Vimentin	53.6	5.06	193	3	P08670		8	
5	40S ribosomal Protein SA	32.9	4.79	212	4	P08865		16	
6	Vimentin	53.6	5.06	49	1	P08670		3	
7	Vimentin	53.6	5.06	321	5	P08670		15	
8	Vimentin	53.6	5.06	128	2	P08670		5	
9	Vimentin	53.6	5.06	508	8	P08670		21	
10	Superoxide dismutase, mitochondrial and a single peptide match to Peroxiredoxin-1	22.3	8.27	35	1	Q06830		5	
11	Transgelin	22.6	8.87	299	5	Q01995		28	
12	Transgelin	22.6	8.87	231	4	Q01995		18	
13	Cathepsin B	38.7	5.88	318	3	P07858		12	
14	Cathepsin B	38.7	5.88	442	4	P07858		17	
15	Ezrin	69.4	5.94	37	1	P15311		1	
16	Pyruvate kinase (PK)	58.4	7.96	167	4	P14618		8	
17	Endoplasmin	92.6	4.76	497	9	P14625		10	
18	Vimentin	53.6	5.06	628	9	P08670		22	
19	Splicing factor, proline- and glutamine-rich	76.2	9.45	39	1	P23246		1	
20	Vimentin	53.6	5.06	202	3	P08670		8	
21	Vimentin	53.6	5.06	156	3	P08670	7		


^a^; Scores greater than 35 are significant and pI; Isoelectric point.

## Discussion

In the present study, we investigated differential proteome profiles between tMSCs derived from low and high grade glioma tumors. These differentially expressed proteins can be classified into three groups: i. Proteins up-regulated in tMSCs derived from both low and high grade glioma tumors, but with a different isoelectric point (pI) and/or molecular weight (different isoforms), ii. Proteins up-regulated only in tMSCs derived from low grade glioma tumors, and iii. Proteins up-regulated only in tMSCs derived from high grade glioma tumors.

Different isoforms of a protein might be generated during alternative splicing at mRNA level or post-translational modification (PMT) at protein level, allowing a single gene to express multiple protein variants possibly with different functions. Abnormal alterations of splicing or PMT by transforming activity, aberrant localization and interaction with other cellular molecules may interfere with normal cellular homeostasis and lead to cancer development (8). We found that different isoforms of two proteins including vimentin and transgelin were differentially expressed in both tMSCs isolated from different gliomas. Vimentin is a member of intermediate filament family. In adults, vimentin expression is limited to connective tissue, MSCs, CNS and muscles. Vimentin overexpression in cancer correlates with increased tumor growth, invasion and poor prognosis. This protein is also considered as a marker of epithelial-mesenchymal transition (EMT). Vimentin has shown to be a target for PTM including citrullination, sumoylation and O-GlcNAcylation modification. Sumoylation of vimentin in the nucleus regulates the structure and motility of glioblastoma multiforme cells. O-GlcNAcylation of glial vimentin is found to prevent hyperphosphorylation of this protein, thus retaining its ability to maintain a rigid structure and provide a scaffold for neuronal migration. Multiple phosphorylation sites on vimentin have been identified, which is associated with functional consequences and assembling of vimentin (9). Different isoforms of vimentin have also been reported in a variety of tissues and tumor types, including six isoforms in pancreatic tumor cell lines, four isoforms in lung tumor cell lines, nine in colon tumor cell lines and 33 in ovarian tumor cell lines (10). Transgelin is an actin-binding protein and mesenchymal cell marker. There are three different isoforms of transgelin, each encoded by a different gene. Both up-regulation and down-regulation of transgelin has been linked to cancer development and progression (11, 12). However in these reports, any possible differential expression of a specific isoform has not been determined. Whether different isoforms of these two proteins play different roles in tMSCs and brain tumors needs more investigations. Increased expression level of very well-known mesenchymal-related proteins, transgelin and vimentin, in the isolated cells in our study again supports the mesenchymal nature of these cells.

Proteins up-regulated in tMSCs derived from low grade glioma tumors were mitochondrial Mn-SOD, 40S ribosomal protein SA, and GTP-binding nuclear protein. Mn-SOD is a member of an antioxidant enzymes family whose main function is to catalyze the superoxide anions in the cytoplasm (13). 40S ribosomal protein, the small subunit of ribosomes in eukaryotic cell, in combination with 60S ribosomal proteins, is involved in protein synthesis. The exact role of these two proteins in brain tumors or mesenchymal cells related to tumors is not clear. Their up-regulation and down-regulation, as well as tumor progressing and suppressing activities have been reported in brain tumors (13-16). GTP-binding nuclear protein Ran is a Ras-related nuclear protein which is required for translocation of proteins through the nucleus of cells. In human glioma cells, it has been shown that paclitaxel-induced cell death was inhibited by Ran suppression (17).

Proteins up-regulated in tMSCs derived from high grade glioma tumors were two isoforms of cathepsin B, endoplasmin, ezrin, peroxiredoxin1, PK, mithochondrial superoxide dismutase, and splicing factor/proline-glutamine rich. The role of the latter protein in brain tumors or tMSCs has not been determined yet.

Cathepsin B, an intracellular protease and a lysosomal enzyme, has been reported to be associated with malignant behavior of several human tumors including colon, breast, prostate, bladder cancer and also glioma (18). One of the initial studies evaluating the role of cathepsin B in glioma progression and invasion was performed by Rempel et al. (19) using several methods (e.g. immune histochemical staining, enzyme activity assays and northern blot analysis) they showed that cathepsin B overexpression is associated with more invasive nature in glioma tumors. On the other hand, it has been shown that up-regulation of cathepsin B was associated with secretion of this enzyme and its cell surface localization. After secretion, cathepsin B attached to the tumor cell surface through the annexin II heterotetramer, and moved to lipid rafts of tumor cells where it could come into contact with serine proteases and matrix metalloproteinase. It has been suggested that pericellular cathepsin B, through its proximity to other proteases in caveolae, takes parts in a proteolytic cascade on the tumor cell surface (20). Endoplasmin, also named glucose-regulated protein 94 (GRP94), is a member of heat shock proteins family, primarily localized in the endoplasmic reticulum. This protein stabilizes and refolds denatured proteins after stresses and increases the cell survival. Endoplasmin is highly expressed in a variety of tumors, including in high-grade glioblastoma, and involved in tumorigenesis by regulating multiple signaling pathways (21). In addition to the localization of endoplasmin in the endoplasmic reticulum, it has also been shown that endoplasmin can be secreted and internalized by other cells (22). Whether cathepsin B and endoplasmin can be secreted from tMSCs and internalized by tumor cells and initiate other signaling pathways in tumor cells needs more investigations.

Ezrin is a member of ezrin-radixin-moesin family proteins whose most important function is cross-linking actin to membrane proteins, thereby regulating cell-cell and cell-extracellular matrix connection and cell motility. It has been shown in several studies that ezrin up-regulation promotes motility and invasion of glioma cells (23). Ezrin overexpression has been observed in a subpopulation of an oral cancer cell line which was CD44+, as a marker of cancer stem cells (24). Ezrin is also shown to increase secretion of other molecules in neutrophils (25). Peroxiredoxin 1, an antioxidant and molecular chaperone, is overexpressed in many cancers including gliomas and its elevation is associated with poor clinical outcome (26, 27). In addition to the ability of secretion of this molecule from tumor cells, it can stimulate secretion of pro-inflammatory cytokines (26). Further investigations are required to determine whether overexpression of ezrin and peroxiredoxin 1 in tMSCs can stimulate the secretion of proglioma molecules from other cells.

PK catalyzes the last step of glycolysis, producing ATP and pyruvate. PK has four isoforms, among of which PK1 is expressed in most tissues, and its spliced variant, called PK2, is shown to be the main isoform expressed in tumors. PK2 expression has been associated with the Warburg effect, which is defined by a high rate of glycolysis for energy production in many cancers, even in the presence of oxygen. PK2 expression levels correlate directly with lactate production in the tumor microenvironment, which is essential for carcinogenesis, tumor growth, and progression (28, 29). PK has been known to be up-regulated in brain tumors including gliomas (30). In our study, isolated tMSCs from high grade gliomas showed overexpression of this enzyme, however further investigations are needed to determine which isoform get involved in this procedure.

Identified proteins in tMSCs obtained from high grade gliomas were those mostly related to the progression of this malignancy, with capability of secretion and internalization by other cells and/or stimulating secretion of other proteins, particularly when they are overexpressed. Most of these molecules have been suggested as a target for cancer therapy. In addition to direct effects on cancer cells, targeting these molecules can influence the harmful effect of adjacent tMSCs on many tumors.

## Conclusion

We cultured MSCs from tumor tissues obtained from patients with low or high grade gliomas, and performed proteomic analysis on these cells for the first time. The identified differentially expressed proteins were related to mesenchymal cells (vimentin and transglin), or related to tumor aggressiveness with a potential of secretory behavior (e.g. cathepsin B). Further investigations are required to clarify the role of the differential expression of these proteins in adjacent tMSCs to glioma tumors.
